# Transcriptome Characterization and Functional Marker Development in *Sorghum Sudanense*

**DOI:** 10.1371/journal.pone.0154947

**Published:** 2016-05-06

**Authors:** Jieqin Li, Lihua Wang, Qiuwen Zhan, Yanlong Liu, Xiaocui Yang

**Affiliations:** College of Agriculture, Anhui Science and Technology University, Fengyang, China; Nazarbayev University, KAZAKHSTAN

## Abstract

Sudangrass, *Sorghum sudanense*, is an important forage in warm regions. But little is known about its genome. In this study, the transcriptomes of sudangrass S722 and sorghum Tx623B were sequenced by Illumina sequencing. More than 4Gb bases were sequenced for each library. For Tx623B and S722, 88.79% and 83.88% reads, respectively were matched to the *Sorghum bicolor* genome. A total of 2,397 differentially expressed genes (DEGs) were detected by RNA-Seq between the two libraries, including 849 up-regulated genes and 1,548 down-regulated genes. These DEGs could be divided into three groups by annotation analysis. A total of 44,495 single nucleotide polymorphisms (SNPs) were discovered by aligning S722 reads to the sorghum reference genome. Of these SNPs, 61.37% were transition, and this value did not differ much between different chromosomes. In addition, 16,928 insertion and deletion (indel) loci were identified between the two genomes. A total of 5,344 indel markers were designed, 15 of which were selected to construct the genetic map derived from the cross of Tx623A and Sa. It was indicated that the indel markers were useful and versatile between sorghum and sudangrass. Comparison of synonymous base substitutions (Ks) and non-synonymous base substitutions (Ka) between the two libraries showed that 95% orthologous pairs exhibited Ka/Ks<1.0, indicating that these genes were influenced by purifying selection. The results from this study provide important information for molecular genetic research and a rich resource for marker development in sudangrass and other *Sorghum* species.

## Introduction

*Sorghum* is a diverse genus consisting of cultivated and wild species, many of which are diploid, possessing considerable genetic and morphological diversity [1]. *Sorghum bicolor* (*S*. *bicolor*), commonly called sorghum, is an important cultivated species in the *Sorghum* genus and is used as food, forage and biofuel [2]. *Sorghum sudanense* (*S*. *sudanense*), commonly called sudangrass, is used as forage for ruminants. It was introduced into the United States in 1909 and rapidly became popular as forage [3]. Now, it is widely cultivated in Russia, Eastern Europe and South Asia [4]. Hybrids of these two species exhibit favorable forage yields, improved quality and disease resistance and therefore, are widely planted in China and the United States [5].

*S*. *bicolor* and *S*. *sudanense* exhibit big differences in morphology [6]. It is expected that genome-wide differences exist between these two species as well, but few studies have been focus on this. In addition, little genetic mechanism research on hybrids between *S*. *sudanense* and *S*. *bicolor* has been conducted due to lack of genomic information of *S*. *sudanense*. Therefore, development of genomic resources for *S*. *sudanense* is the basis of molecular biology studies on *S*. *sudanense* and hybrids of *S*. *bicolor* and *S*. *sudanense*.

RNA-sequencing (RNA-Seq) is a powerful tool for transcription profiling, providing a rapid access to a collection of expressed sequences (transcriptome). RNA-Seq offers novel opportunities in functional genomics and for developing molecular markers in non-model plants [7]. Up to now, RNA-Seq has been successfully applied in different domains of life from yeast to plants [8,9,10].

In this study, the leaf transcriptomes of two *Sorghum* species, *S*. *bicolor* and *S*. *sudanense*, were characterized. The main objective was to compare the transcriptomes of the two *Sorghum* species, and to develop DNA markers for genetic mapping of the genes for traits specific to *S*. *sudanense*. In addition, substitution rates of orthologous transcript pairs were estimated to calculate functional sequence diversity.

## Materials and Methods

### Plant materials

Sorghum Tx623B and sudangrass S722 were grown and maintained in a greenhouse under natural daylight condition. When the plants grew to the seven-leaf stage, all the visible leaves of three biological replicates were taken, frozen in liquid N_2_ and stored at -80°C until further use. Each replicate involved three plants.

### RNA extraction and Illumina sequencing

Total RNA was extracted using an RNA Prep Pure Plant kit (LabKit Co., China) and analyzed for quality and quantity by an Agilent 2100 Bioanalyzer (Agilent Technologies, Santa Clara, CA, USA). Equal quantities of total RNA from the three biological replicates were pooled before used for cDNA library construction and sequencing. cDNA libraries were constructed using the Illumina RNAseq kit (Illumina, USA). Solexa adapters were then ligated to the ends of the cDNA fragments for Solexa sequencing. High-throughput sequencing was performed using Illumina Hiseq 2500 (Illumina, USA). Reads were 125 bases in length and generated from each end of the DNA fragments in paired-end sequencing.

### Gene annotation and differential expression analysis

The adaptor sequences and low-quality sequence reads were removed from the data sets. Raw sequences were transformed into clean tags after data processing. These clean tags were then mapped to the reference genome sequence (http://phytozome.net/, *Sorghum bicolor* v2.1). GO (Gene Ontology, http://www.geneontology.org/) and COG (Clusters of Orthologous Groups of proteins, http://www.ncbi.nlm.nih.gov/COG/) analyses were subsequently performed.

The expression level of each gene was measured as the normalized number of matched clean tags. The normalization method of reads per kilobase per million mapped (RPKM) was used in this study [11]. The IDEG6 software was used to test the significance of gene expression. The false discovery rate (FDR) method was used to tune the threshold *P* value. An FDR <0.01 and an absolute log_2_ ratio ≥1 were used to judge the significance of differences in gene expression.

### SNP and indel detection

All S722 reads were used to mine SNP markers by aligned to the sorghum reference genome using TopHat [12]. SNP loci were detected using SAMtools between the library and the reference genome [13]. For the nucleotides to be counted as a SNP, the following parameters were required: (1) The sequencing depth was more than 5X, but less than 100X; (2) the quality value of variant calling was larger than 20%; and (3) the distance between SNP loci was longer than 5 bases.

Indel loci were detected using GATK by aligning S722 reads to the reference genome using STAR. The parameters for indel loci detection were as follows: (1) The quality value was larger than 20; and (2) the distance between detected indel loci was longer than 35 bases.

### Indel marker design and genetic map construction

All indel sequences (500 bases) were extracted from the sorghum reference genome. Then, indel markers were designed using Primer 3.0 [14]. The parameters were as follows: (1) T_m_ value was 55–60°C; and (2) PCR target product was 150–350 bases. A hundred indel markers (more than 3 bases of difference) were synthesized by the Springen Biotechnology Company (Nanjing, China). A hundred indel markers (10 markers for each chromosome) were screened for polymorphism between Tx623B and S722 by size detection using 8% non-denaturing polyacrylamide-based electrophoresis. To prove the value of these indel markers, a set of 92 F_2_ individuals derived from a cross between sorghum Tx623A and sudangrass Sa was used as the mapping population. Then, 15 indel markers were used to construct the genetic map. The primers are listed in S1 Table. The genetic map was constructed using Windows QTL IciMapping version 4.0 [15]. The Kosambi map function was used to calculate the genetic distances.

### Analysis of non-synonymous and synonymous mutations

All reads were aligned to the reference genome for coding frame prediction using ClustalW2. The ratio of non-synonymous to synonymous mutations (Ka/Ks) was calculated for these coding reads using PAML_yn00 program. The related parameters for PAML_yn00 program were verbose = 0, icode = 0, weighting = 1, commonf3x4 = 0 and ndata = 1.

## Results

### Transcriptome sequencing output and aligning to the reference genome

High quality sequencing data is the base of transcriptome analysis. RNA-Seq of the two cDNA libraries, Tx623B and S722, was performed. The Illumina sequencing generated 48,155,337 sequence reads and 9.62 Gb of sequence data. Both GC contents of the sequences of the two libraries were larger than 50% (58.26% and 58.15% for Tx623B and S722, respectively), and CycleQ20 was 100%. All these values indicated that the accuracy and quality of the sequence data were high enough for further analysis.

All reads were aligned to the *S*. *bicolor* genome which contained 33,472 gene models. The results showed that most reads of the two libraries (88.79% and 83.88% for Tx623B and S722, respectively) matched to the *S*. *bicolor* genes (Table 1). The majority of the mapped reads were “perfectly mapped reads” without any base-pair mismatch or indel. Totally, 21,572 and 20,752 genes were identified in the reads of the Tx623B and S722 libraries, respectively. The results showed that more than 60% putative genes in the reference genome were expressed in the seven-leaf stage of the two *Sorghum* species.

**Table 1:** RNA-sequencing results for sorghum Tx623B and sudangrass S722.

	Tx623B	S722
Total reads	24,257,222	23,898,115
Total base	4.85 Gb	4.77 Gb
GC (%)	58.26	58.15
CycleQ20 (%)	92.90	93.03
Mapped reads	43,077,834 (88.79%)	40,089,957 (83.88%)
Perfectly mapped reads	26,780,019 (62.17%)	39,373,636 (53.32%)
Mismatched reads	5,881,880 (13.66%)	8,354,470 (20.84%)
Indel reads	8,825,105 (20.49%)	7,949,694 (19.83%)
Indel and mismatched reads	1,590,830 (3.6%)	2,408,455 (6.01%)
Identities	98.60%	98.21%

### Differentially expressed gene analysis and new gene analysis

**Fig 1:**
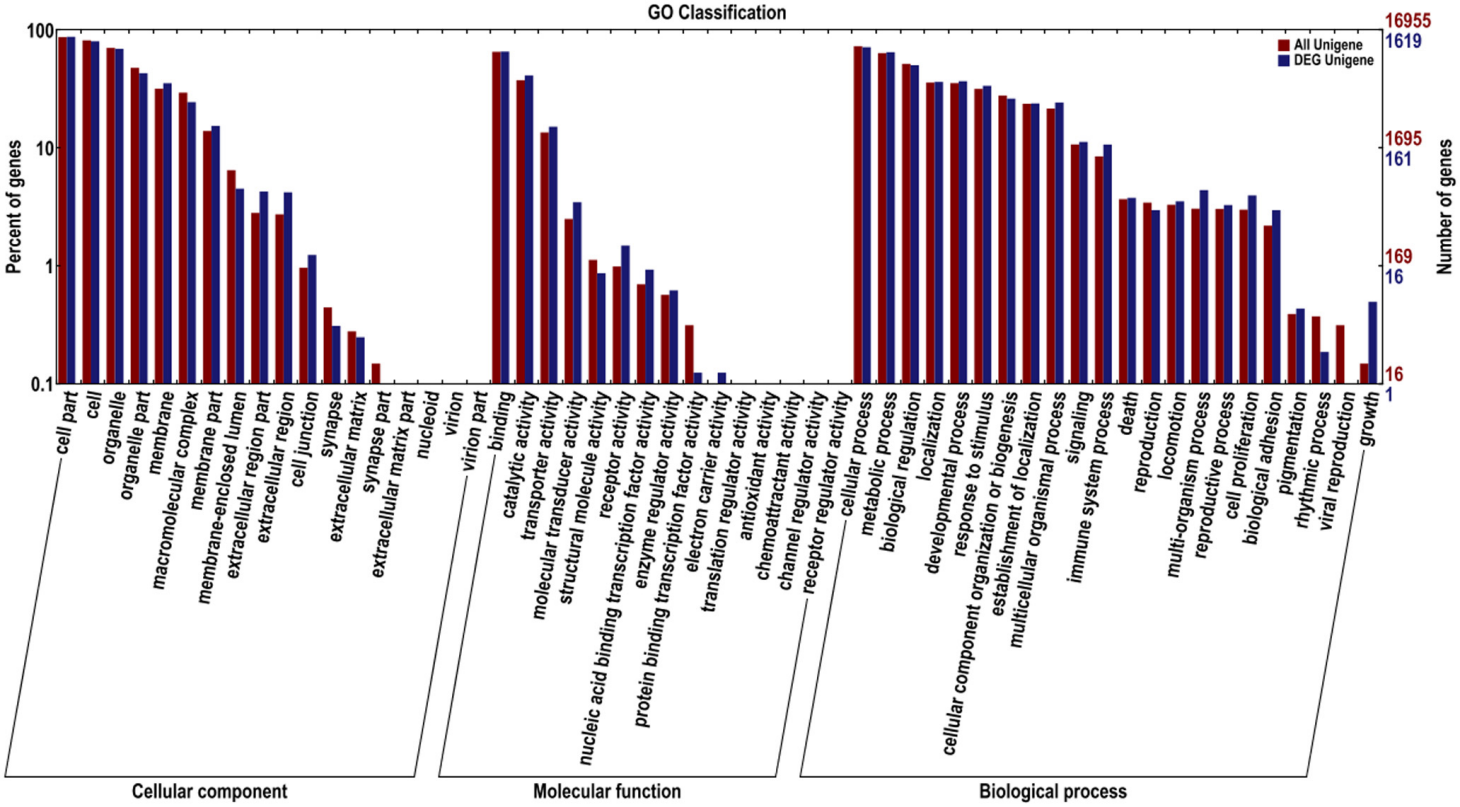
Functional annotation of sequences based on Gene Ontology (GO) categorization.

A total of 2,397 differentially expressed genes (DEGs) were detected by RNA-Seq between the two libraries, including 849 up-regulated ones and 1,548 down-regulated ones (S2 Table). The results showed that there were wide differences in gene expression between the two *Sorghum* species. And most of the DEGs were down-regulated in *Sorghum sudanense*. To get annotations, the DEGs of the two libraries were mapped using the GO database. After GO analysis, the 1,619 unigenes were divided into three groups: molecular function, cellular component and biological process (Fig 1). Then, gene set enrichment analysis was applied to unveil that common unigenes and DEG unigenes mainly differed in protein transcription factor activity, rhythmic process, viral reproduction and growth. These results explained the difference in growth rate between *S*. *bicolor* and *S*. *sudanense* in the seven-leaf stage.

**Fig 2:**
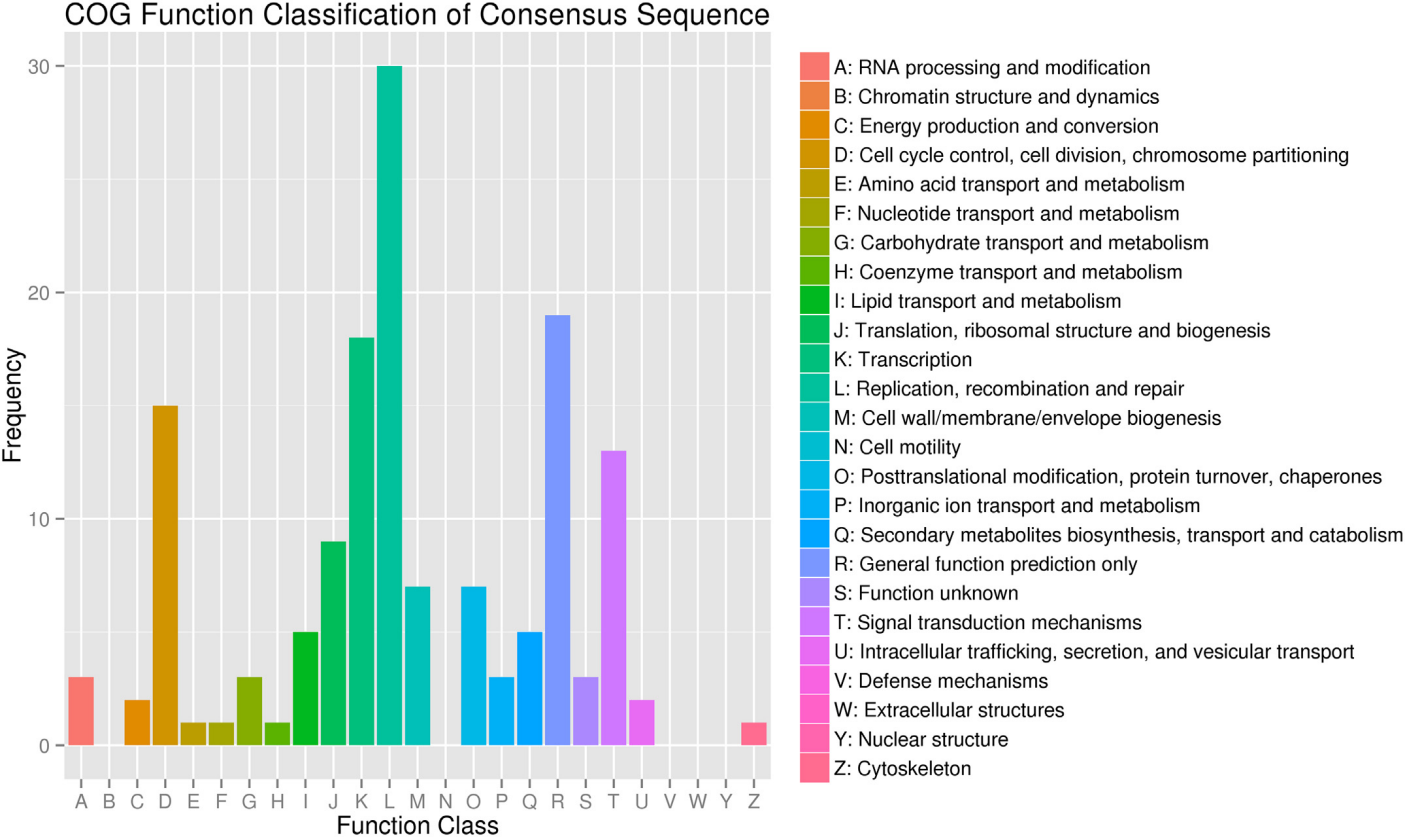
Functional classification of the new genes using COG.

A total of 1,470 new genes were identified in S722 (S3 Table). These new genes, of different lengths, were annotated using different databases. Totally, 102 new genes were categorized into 25 functional groups according to the COG classification (Table 2). The group‘replication, recombination and repair’ contained the highest number of new genes, followed successively by the groups of ‘general function prediction only’, ‘transcription’, ‘cell cycle control, cell division, chromosome partitioning’ and ‘signal transduction mechanism’ (Fig 2). The percentage of annotated new genes was 60.6%. The results indicated that further analysis of the unannotated new genes may shed light on the genetic basis for the morphological differences between sorghum and sudangrass.

**Table 2:** The 102 new genes annotated using different annotation databases.

Anno_Database	Annotated_Number	300≤Length<1000	Length≥1000
COG_Annotation	102	23	78
GO_Annotation	425	125	299
nr_Annotation	876	287	584
All_Annotated	891	290	596

### SNP and indel discovery and validation

SNP and indel loci are important information to develop markers for molecular research of sorghum, sudangrass and hybrids of sorghum and sudangrass. A total of 44,495 SNPs were discovered by aligning S722 reads to the reference genome (S4 Table). The highest and lowest number of SNPs was found on chromosome 1 and 5, respectively (Table 3) with a mean of 4,415. This indicated that every 15.7 Kb of the sudangrass genome had 1 SNP between *S*. *bicolor* and *S*. *sudanense*, which was good enough for developing markers. Of these SNPs, 33,751 were located in genes, while 10,744 were in intergenic regions. It was found that more than 70% of the SNPs could be developed into functional markers. Further analysis showed that 61.37% of the SNPs were transition, and the value was almost the same among different chromosomes.

**Table 3:** SNPs identified in *Sorghum sudanense* S722.

Chromosome	SNP_number	SNP_number_gene	SNP_number_intergenic	Transition (%)	Transversion (%)
Chromosome_1	6,965	5,545	1,420	61.84	38.16
Chromosome_2	5,528	4,335	1,193	61.03	38.97
Chromosome_3	5,438	4,308	1,130	59.07	40.93
Chromosome_4	5,401	4,203	1,198	61.78	38.22
Chromosome_5	2,965	2,010	955	60.20	39.80
Chromosome_6	4,132	3,140	992	61.79	38.21
Chromosome_7	2,830	2,052	778	62.30	37.70
Chromosome_8	3,287	2,350	937	61.15	38.85
Chromosome_9	3,649	2,668	981	61.61	38.39
Chromosome_10	3,957	2,958	999	63.58	36.42
Super_10	343	182	161	60.79	39.21
Total	44,495	33,751	10,744	61.37	38.63

**Fig 3:**
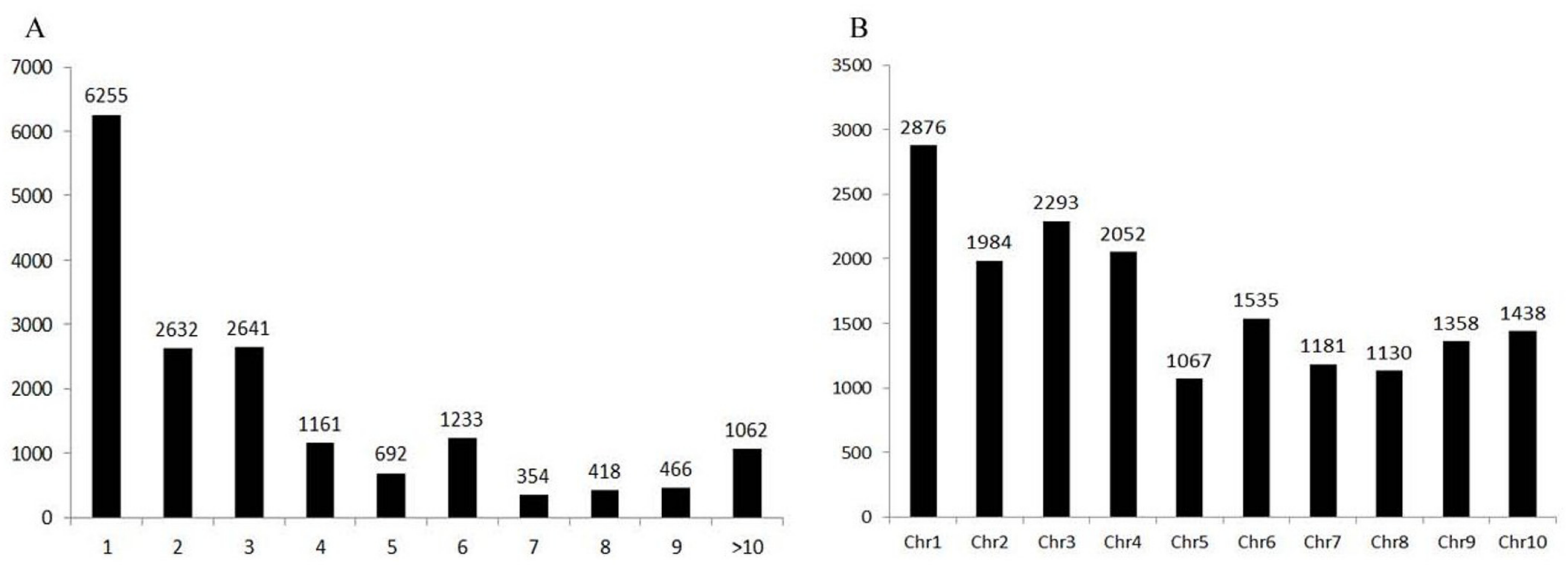
The distribution of indel loci in sorghum (A: the distribution of base differences, X axis is base difference, and Y axis is number; B: the distribution of indel in different chromosomes, X axis is chromosome, and Y axis is number).

Indel markers are ubiquitous, co-dominant and locus-specific [16]. They are a type of useful marker in molecular genetics. In this research, 16,928 indel loci were identified by aligning S722 reads to the sorghum reference genome (S5 Table). On average, one indel was found in every 41.3 Kb of the sudangrass genome. Of these indels, most were only one-base different between *S*. *bicolor* and *S*. *sudanense*; 68.1% were 1- to 3-base different (Fig 3A). The highest and lowest number of indel was found on chromosome 1 and 5, respectively (Fig 3B).

### SNP and indel validation and genetic map construction

To validate the SNPs, 30 SNP loci were randomly selected to be sequenced using the Sanger sequencing method. The amplification primers are listed in S6 Table. The results confirmed that all the SNP loci should be reliable.

**Fig 4:**
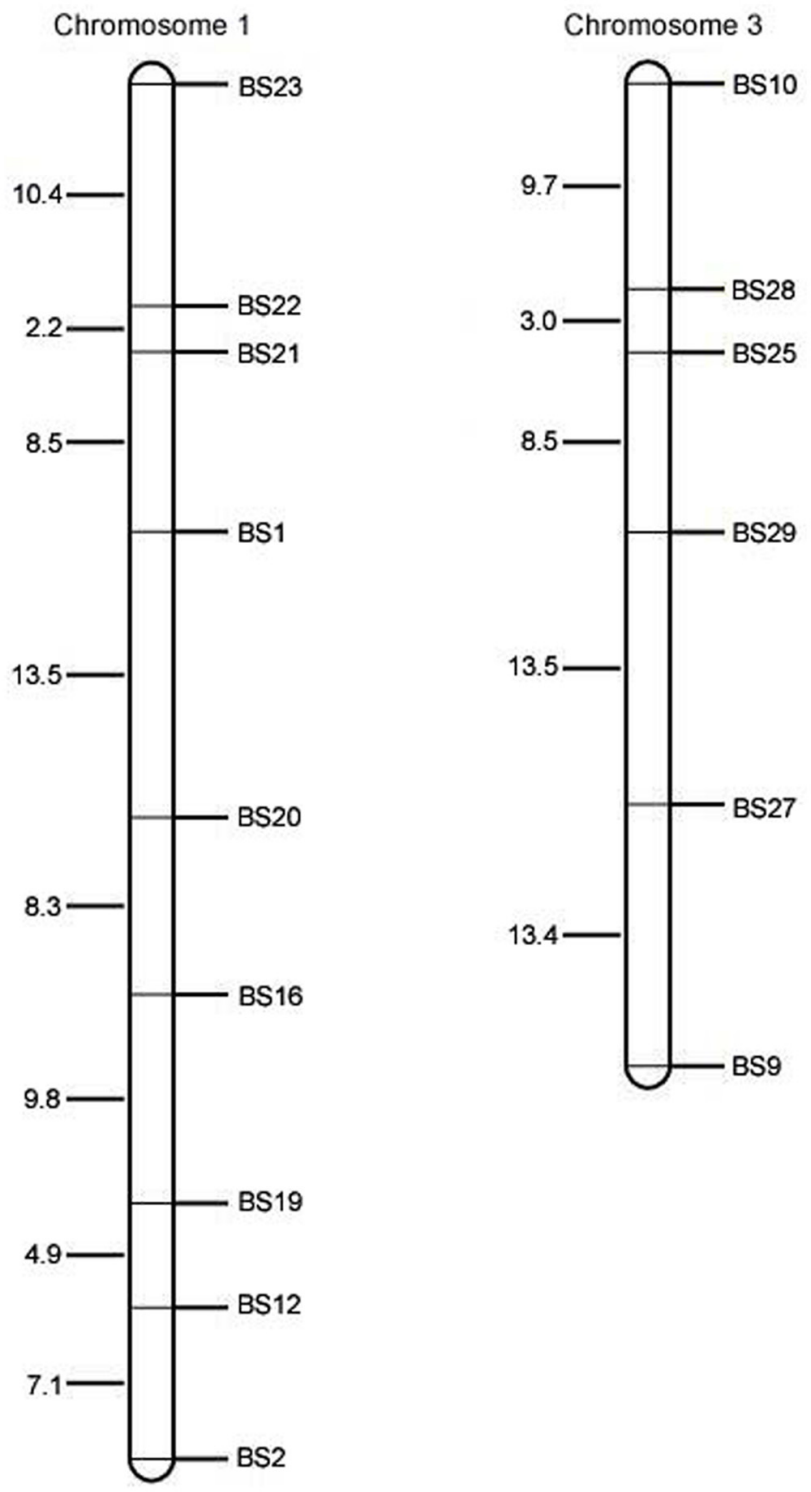
The genetic map of the F_2_ population derived from the cross of sorghum Tx623A and sudangrass Sa.

A total of 5,344 indel primers were designed for the 16,928 indel loci using Primer 3.0 (S7 Table), 100 of which (more than 3 bases of difference) were synthesized and screened between Tx623B and S722. It turned out that 5 did not amplify PCR product. Ninety-five indel markers showed polymorphism between the two accessions. The percentage of polymorphism was 82% between Tx623A and Sa, further proving the universality of the 100 indel markers. Then, 15 out of the 100 indel markers were selected to construct the genetic map of the F_2_ population derived from the cross of Tx623A and Sa (Fig 4). All the results indicated that the SNPs and indels could provide important information for marker mining and applications in molecular genetic research and molecular marker-assisted breeding.

### Synonymous and non-synonymous mutation analysis

**Fig 5:**
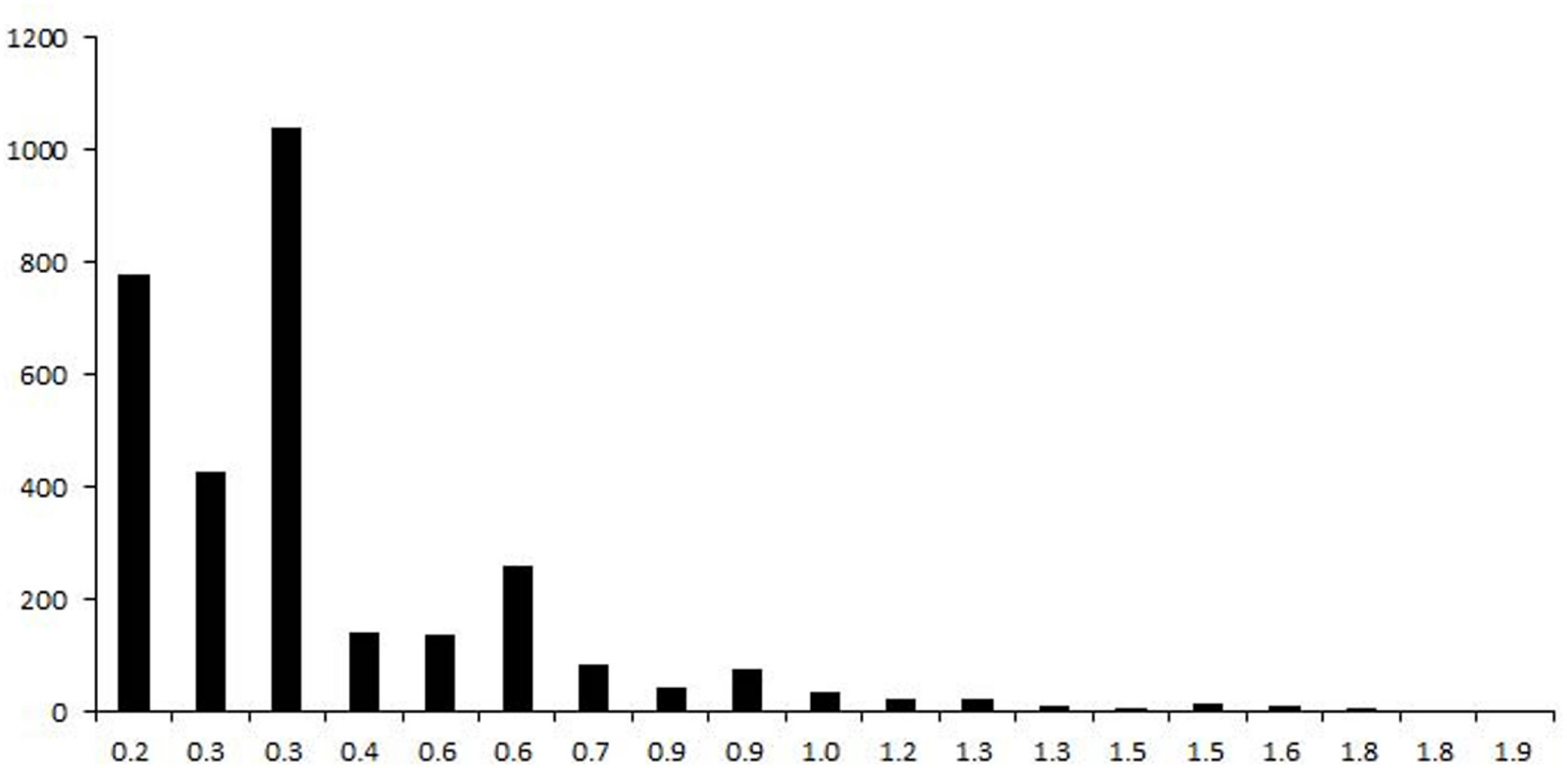
A histogram of Ka/Ks distribution.

To detect functional sequence diversity, putative exon sequences were compared between S722 and the reference genome. Comparison between synonymous base substitutions (Ks) and non-synonymous base substitutions (Ka) can tell sequences are influenced by purifying or diversifying selection. The average Ka/Ks value was 0.39 for orthologous pairs. About 95% of the orthologous pairs exhibited a Ka/Ks<1.0, indicating that the genes were influenced by purifying selection (Fig 5). And 5% of the orthologous pairs showed a Ka/Ks>1.0, indicating that the genes were influenced by diversifying selection. Functional annotation was performed for the 15 pairs under diversifying selection. The largest numbers of annotated pairs were of catalytic activity and DNA/RNA binding molecular functions (S1 Fig).

## Discussion

Transcriptome sequencing provides a new tool for genomic studies on model or non-model organisms [10,17]. In this study, the transcriptome of sudangrass, which is an important forage in temperate regions, was characterized. Compared to sorghum, sudangrass has a higher forage yield and a higher growth rate [5]. So, there is a need to understand the genetic mechanisms underlying the phenotypic differences between *S*. *bicolor* and *S*. *sudanense* so that *S*. *sudanense* genes can be utilized to develop related breeding strategies. Learning the expression profiles of *S*. *bicolor* and *S*. *sudanense* and the sequence polymorphisms provides an opportunity to understand the genetic basis of phenotypic differences. In this research, some up-regulated transcriptional factors involved in plant growth and development were also identified. For example, the up-regulated *Sorghum_newGene_2588* in sudangrass was annotated as a Myb-related transcriptional factor involved in plant growth and development [18].

It is controversial about whether *S*. *bicolor* and *S*. *sudanense* are actually of a same species. Snowden treated sudangrass as *S*. *sudanense*, a species different from sorghum in spikelet, anthotaxy and plant traits [19]. De Wet suggested that sudangrass be placed as a subspecies, *drummondii* (steud), of *S*. *bicolor* (L.) Moench [20]. The results from this study showed that the GC contents of sorghum Tx623B and sudangrass S722 were just slightly different. The percentages of the mapped reads aligned to the reference genome also differed only slightly between the two transcriptomes. All these data indicated a high genomic similarity between the two transcriptomes. Although the results of this research could not support that sudangrass should be placed as a subspecies of *S*. *sorghum*, they provided a clue for further studies on the place of sudangrass in taxonomy.

The polymorphism of SSR markers developed from the sorghum genome was low (19.48%) between sorghum and sudangrass [21]. Therefore, it is important to develop high diversity markers for genetic research on sudangrass and hybrids of sorghum and sudangrass. As indel markers within genes are functional markers, they are superior to random DNA markers, such as RAPD, SSR, and AFLP, because functional markers are completely linked with trait locus alleles [22]. A total of 16,928 indel loci between S722 and the reference genome were detected, and 5,344 indel markers were developed. The percentage of polymorphism was 95% in the 100 selected indel markers between Tx623B and S722; the percentage of polymorphism was 82% between Tx623B and Sa. It was indicated that the indel loci were reliable and the indel markers were diverse between sorghum and sudangrass. To further verify the value of these indel markers, they were used to construct the genetic maps of chromosomes 1 and 3 derived from a cross between sorghum Tx623A and sudangrass Sa. The results demonstrated that these indel markers developed will be useful for QTL mapping and molecular-assisted breeding in sorghum, sudangrass, and hybrids of sorghum and sudangrass.

SNP detection is an important part of molecular genetic research because SNP loci can be exploited to construct high-density genetic maps and genome-wide association studies [8,23,24]. A total of 44,495 SNP loci between S722 and sorghum genome were detected in this work. The frequencies of transition and transversion were comparable to those observed in other plant species [25, 26, 27]. The density of SNP loci was 2.6 times that of indel loci. Therefore, SNP loci are an important supplement to indel markers in marker development for construction of high-density genetic maps or gene mapping. Additionally, it was found that 24.3% of the SNP loci were genic. These genic SNP loci will be more appropriate for developing phenotype-associated markers.

The ratio of Ka to Ks provides a measure of the selection pressure to which a gene pair is subjected and has been used to tell whether a gene is under positive/diversifying selection or under negative/purifying selection. Our results are consistent with the findings by other researchers that most orthologous genes are under purifying selection. It was also suggested that positive selection pressure results in the maintenance of divergence between the two species, which is also observed in other organisms [25, 28].

## Conclusion

In this study, the transcriptome of *Sorghum sudanense* was sequenced and characterized using the next-generation sequencing platform. A total of 2,397 DEGs were detected by RNA-Seq between the two libraries, including 849 up-regulated ones and 1,548 down-regulated ones. A total of 44,495 SNPs were discovered by aligning S722 reads to the sorghum reference genome. A total of 16,928 indel loci were identified between the two genomes. A total of 5,344 indel markers were designed, 15 of which were selected to construct the genetic map derived from the cross of Tx623A and Sa. This work provides a set of functional markers and a resource for mapping of and molecular genetic research on sudangrass and other *Sorghum* species.

## Supporting Information

S1 Fig GO Classification of orthologous pairs. (DOCX)

S1 Table The primers used to construct the genetic map. (DOCX)

S2 Table Differentially expressed genes between the two libraries. (XLSX)

S3 Table New gene sequences. (XLSX)

S4 Table SNP information. (XLSX)

S5 Table Indel loci information. (XLSX)

S6 Table The primers for SNP validation. (DOCX)

S7 Table Indel primers. (XLSX)
