# Supplementary figures and images for: Transcriptome Characterization and Functional Marker Development in Sorghum Sudanense
Source: PLoS One. 2016 May 6;11(5):e0154947. doi: 10.1371/journal.pone.0154947 (PMC4859472; doi:10.1371/journal.pone.0154947)

Figure S1 Go Classification of orthologous pairs Ka/Ks>1
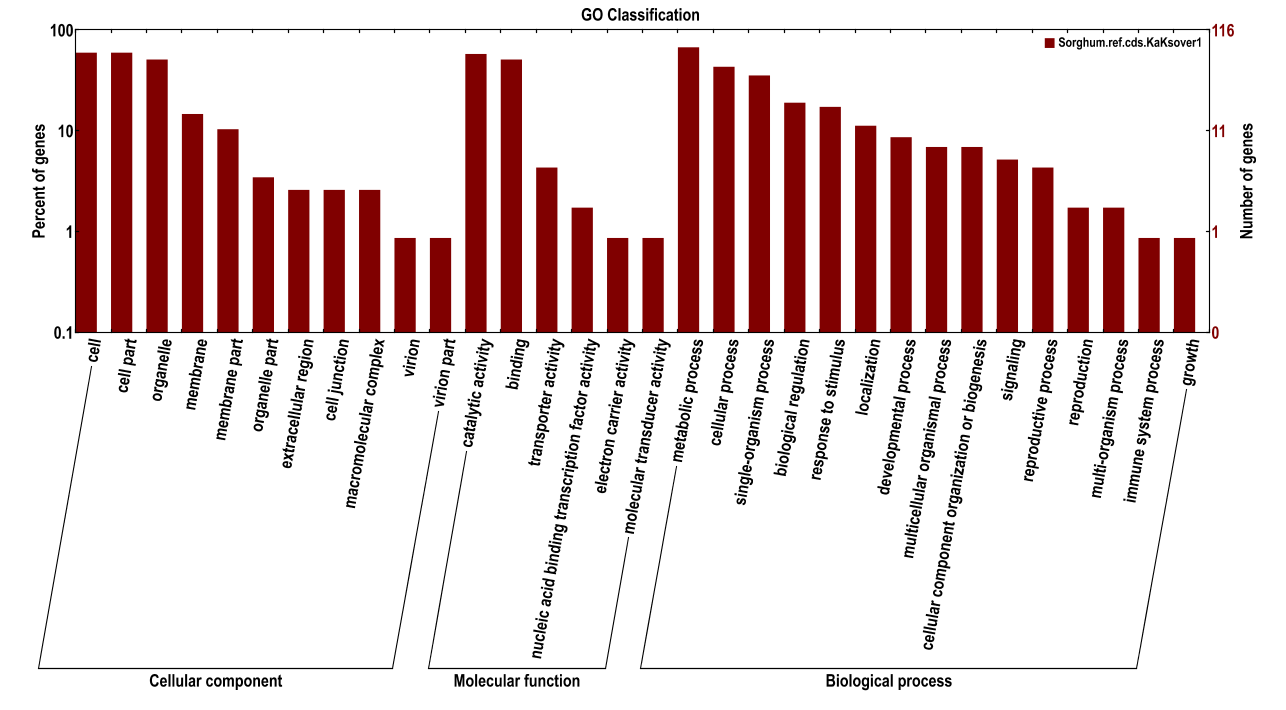

Supplement: S1 Fig — (DOCX) [file pone.0154947.s001.docx]
