# Supplementary material for: Transcriptome Characterization and Functional Marker Development in Sorghum Sudanense
Source: PLoS One. 2016 May 6;11(5):e0154947. doi: 10.1371/journal.pone.0154947 (PMC4859472; doi:10.1371/journal.pone.0154947)
Supplement: S1 Table — (DOCX) [file pone.0154947.s002.docx]

| Primer name | Chromosome | Indel  Location | Forward sequence(5’-3’) | Reverse sequence(5’-3’) |
| --- | --- | --- | --- | --- |
| BS23 | 1 | 17704525 | CCCCTACGACTACATCGA | AACGAGCCATCCAACAGG |
| BS22 | 1 | 272651 | CCTCTTCCTCTACCGCACAA | ATCGGACTTCCTTCCTTTCC |
| BS21 | 1 | 341890 | AAGGCGAAGGCTGAAGAG | AATGTGGAGGGCGAAGAT |
| BS1 | 1 | 3795727 | GACGACGAGGAAGATGAA | CTCCGAGAAGAGCTTGTTAG |
| BS20 | 1 | 7370927 | GGGAGATCGGATTTGCAG | CTTCGTGGCTCGTGGGTA |
| BS16 | 1 | 15799310 | CCATACCCATCCAAATCC | AGTTCCACCTGGAGCAGA |
| BS19 | 1 | 45774808 | TTTGTCTTGGGCGTGGAA | GGGAAACGTCGTTGGGAT |
| BS12 | 1 | 65109422 | GGAGTCGTCGATTTCTATC | TTCAAACGGAGTGTTCAT |
| BS2 | 1 | 73612807 | ACAGTTTGCCTGTAAATC | AGTACTTCCTCCGTTCAT |
| BS10 | 3 | 218699 | AACCCAAACCTAAGAGTA | GATTCCCAATCAGATGTA |
| BS28 | 3 | 2895596 | GAAGCCTCGACGAAGCAG | TGGTGAAACGACTAACGGTGT |
| BS25 | 3 | 42148435 | CATGCCTGTTTCCATACC | AAATTGCCTCGTAGTTGG |
| BS29 | 3 | 64523779 | CCCACAACGCCGAACTCT | GACGACCGATTCCTCCCT |
| BS27 | 3 | 74370120 | GAAGCCAACTAAGGGATACGG | AGGACGCCTTCAAGCACA |
| BS9 | 3 | 57314217 | GGCTTGTCGGCATTAGCG | TGCAGGGCGTATGGCTTT |
