# Supplementary material for: Transcriptome Characterization and Functional Marker Development in Sorghum Sudanense
Source: PLoS One. 2016 May 6;11(5):e0154947. doi: 10.1371/journal.pone.0154947 (PMC4859472; doi:10.1371/journal.pone.0154947)
Supplement: S6 Table — (DOCX) [file pone.0154947.s007.docx]

Table S5 The primers of SNP validation

| No. | Chromosome | position | Forward primer | Reverse primer |
| --- | --- | --- | --- | --- |
| 1 | chromosome_1 | 156366 | TGCCAAATCAGTGCCATCT | CAGTATCGTCGGGCGTGT |
| 2 | chromosome_1 | 65806933 | CTGCGAGGAGCCGTTGTTC | CACGACTTCAGCCGCGTTC |
| 3 | chromosome_1 | 62415064 | GCAGAAGAAGAAGAGGGAG | ACGAGGAGGTCACCAGAA |
| 4 | chromosome_2 | 1217178 | GTTCGTGGGTACATCTCG | CTTTGGTTCCGACTGATT |
| 5 | chromosome_2 | 5657474 | TCGCAGTTTGTGGTTCAG | AGTCCTAATGCCCAGAGG |
| 6 | chromosome_2 | 77865449 | GGGGTTCTTCATGGTGCCG | CAAGGCCAAGCGCAAGCT |
| 7 | chromosome_3 | 1006075 | AAACTGAGCAGCAAGGGTTAC | TGTTCCGATGCTTGAAGTGC |
| 8 | chromosome_3 | 4648831 | GCCGCGTATACCTCGTTGC | CGCTCGCCGTCCTCTACC |
| 9 | chromosome_3 | 70641292 | CGCACCTCGCGTGGTAAA | TGCCGGGAAAGAAACATC |
| 10 | chromosome_4 | 145678 | ATCAACGAGGTTGGCGAGTG | GCAGCTCTGCGAGATGCTCT |
| 11 | chromosome_4 | 1594891 | ATCAACGAGGTTGGCGAGTG | GCAGCTCTGCGAGATGCTCT |
| 12 | chromosome_4 | 60386007 | TCGGACATTGCTGATTGA | TTTGACAGATGACGTGGA |
| 13 | chromosome_5 | 2499982 | GGGATCAGGAGCAAACAG | CCGCCAGTGGAATAAGAT |
| 14 | chromosome_5 | 58018167 | CATCGCCCGCCAGGTTCA | GGCTTCGTTCGGCGTCTCG |
| 15 | chromosome_5 | 60762209 | TTTTGAGGATGGGAGGAG | TGGGTGTAGGAAGGGAGA |
| 16 | chromosome_6 | 41058206 | TTCAACTTGCTGGAACAT | AACAAAAGAAGGCAAAGG |
| 17 | chromosome_6 | 46638100 | CGCCACAGACAATGCTAC | ACATCAGTTTTCGAGGGA |
| 18 | chromosome_6 | 50123822 | GTTTGTTCCCTTTGAGTT | CTGATAGTCTTCCCACCT |
| 19 | chromosome_7 | 3863595 | TGATGAATCACAAGGACCAA | GAAGGAAGTGCGAGGAAA |
| 20 | chromosome_7 | 51788497 | CTCAAAGCCTCAAATACA | GCACAGTCAATCGGAAAA |
| 21 | chromosome_7 | 62865766 | AATACAAGATACACGCTCCT | GTTGCTGACTTGCCACTA |
| 22 | chromosome_8 | 2517112 | TAAGCACGAACTAGCACCA | CGCAGCCTCTGATCTGTT |
| 23 | chromosome_8 | 53888711 | ATGGCGACGAGCTGGGTGAC | TTTGCTTCCCTTCCGTGGTG |
| 24 | chromosome_8 | 55365174 | TTTACTTGTATCGCTCTTG | AGCTACTCGTAGGCACAG |
| 25 | chromosome_9 | 1012816 | ACAAGAAGACACGAGCCATT | CAACTGTTTCAGCGCCAT |
| 26 | chromosome_9 | 9233959 | GCCACAAACCTCCCTCCTCC | TCATCCGCCTCTTCGTCCC |
| 27 | chromosome_9 | 51141426 | GGCGATGATCGCTTCGTT | CGTGCGTTTCCTTTTGTTTC |
| 28 | chromosome_10 | 22552418 | CGAGACGACGATAGCAGC | CAAGACCCATTCGGATGTA |
| 29 | chromosome_10 | 38778834 | TCAAGAGGCACCGTACAAG | GTGGCAAGAACACGCAGT |
| 30 | chromosome_10 | 52135654 | ACAAGATTGACCAGGAACT | AATTGTCTTACTGGGAGTTT |
